# Supplementary material for: Multiplex Real-Time PCR Assay for Six Major Carbapenemase Genes
Source: Pathogens. 2021 Mar 1;10(3):276. doi: 10.3390/pathogens10030276 (PMC7999841; doi:10.3390/pathogens10030276)
Supplement: Supplementary file 1 [file pathogens-10-00276-s001.pdf]

	10^3 copies/ 15µl		10^2 copies/ 15µl		2×10^1 copies/ 15µl		10^1 copies/ 15µl		8 copies/ 15µl	
	C	Log	C	Log	C	Log	C	Log	C	Log
**NDM**										
1	976	2.99	96	1.98	14	1.14	10	1.00	11	1.04
2	1080	3.03	144	2.16	13	1.12	13	1.11	14	1.13
3	1080	3.03	80	1.90	10	0.99	5	0.71	10	0.99
4	1000	3.00	75	1.87	9	0.97	10	1.02	3	0.41
5	963	2.98	81	1.91	13	1.12	7	0.85	11	1.06
mean	1020	3.01	95	1.97	12	1.07	9	0.94	10	0.93
C.V.	5.54	0.80	29.85	5.86	17.68	7.47	33.11	16.74	43.13	31.41
**KPC**										
1	1010	3.00	105	2.02	19	1.29	9	0.97	8	0.92
2	984	2.99	113	2.05	26	1.41	30	1.47	10	0.98
3	993	3.00	107	2.03	41	1.61	16	1.19	5	0.74
4	1000	3.00	102	2.01	21	1.33	15	1.17	10	1.00
5	939	2.97	87	1.94	29	1.45	14	1.16	10	1.01
mean	985	2.99	103	2.01	27	1.42	17	1.19	9	0.93
C.V.	2.79	0.41	9.36	2.11	30.91	8.85	45.66	15.16	22.65	12.24
**IMP**										
1	978	2.99	119	2.08	20	1.30	5	0.71	13	1.10
2	984	2.99	133	2.12	15	1.17	16	1.20	2	0.26
3	1030	3.01	100	2.00	19	1.27	5	0.70	7	0.85
4	1000	3.00	109	2.04	19	1.28	13	1.10	5	0.73
5	1000	3.00	135	2.13	16	1.30	11	1.05	13	1.10
mean	998	3.00	119	2.07	18	1.24	10	0.95	8	0.81
C.V.	2.02	0.29	12.67	2.69	12.63	4.51	48.01	24.38	59.50	42.82
**GES**										
1	1050	3.02	66	1.82	20.00	1.30	11.20	1.05	9.45	0.98

Supplementary Table 1. Reproducibility of the multiplex quantitative real-time PCR assay using DNA standards

of

2	962	2.98	82	1.91	21.10	1.32	11.00	1.04	5.71	0.76
3	1040	3.02	71	1.85	23.20	1.37	4.92	0.69	8.96	0.95
4	1000	3.00	63	1.80	19.00	1.28	7.85	0.89	6.5	0.81
5	994	3.00	70	1.84	16.50	1.22	5.27	0.72	11.4	1.06
mean	1009	3.00	70	1.84	20	1.30	8	0.88	8	0.91
C.V.	3.56	0.52	10.12	2.32	12.45	4.24	37.37	19.30	27.43	13.51
**OXA**										
1	1000	3.00	109	2.04	20.00	1.30	5.80	0.76	6.1	0.79
2	958	2.98	102	2.01	29.30	1.47	6.44	0.81	6.56	0.82
3	1010	3.00	117	2.07	17.50	1.24	5.00	0.70	9.19	0.96
4	1020	3.01	108	2.03	24.30	1.39	9.50	0.98	9.17	0.96
5	966	2.98	112	2.05	18.30	1.26	11.00	1.04	9.18	0.96
mean	991	3.00	110	2.04	20	1.33	7.5	0.86	9	0.90
C.V.	2.76	0.40	5.02	1.07	31.13	7.00	50.98	16.95	15.37	9.94
**VIM**										
1	1050	4.99	66	1.82	19.20	1.28	18.60	1.27	17.5	1.24
2	962	2.98	82	1.91	26.40	1.42	15.30	1.18	2.93	0.47
3	1040	3.02	71	1.85	20.00	1.30	14.10	1.15	7.14	0.85
4	1000	3.00	63	1.80	20.20	1.31	10.80	1.03	7.23	0.86
5	994	3.00	70	1.84	18.50	1.27	11.20	1.05	4.61	0.66
mean	1009	3.00	70	1.84	21	1.32	14	1.14	8	0.82
C.V.	3.56	0.52	10.12	2.32	15.20	4.65	22.85	8.62	71.97	35.18

NDM, KPC, IMP, GES, OXA, and VIM.

Five different DNA concentrations (8, 10^1, 2×10^1, 10^2, 10^3 copies/ 15 µL) were prepared and repeatability of the test was confirmed with five times overlapping experiments. In all the experiments, Cp (crossing point) values were confirmed to be under 40 or less. C, copies/15µL. Log, Log10 copies/15µL. C.V., coefficient of variation (%).
